# Building health system capacity to improve maternal and newborn care: a pilot leadership program for frontline staff at a tertiary hospital in Ghana

**DOI:** 10.1186/s12909-019-1463-8

**Published:** 2019-02-11

**Authors:** Erin Pfeiffer, Medge Owen, Christie Pettitt-Schieber, Romeck Van Zeijl, Emmanuel Srofenyoh, Adeyemi Olufolabi, Rohit Ramaswamy

**Affiliations:** 1 Kybele, Inc., Lewisville, USA; 2 Wake Forest School of Medicine, Winston-Salem, USA; 3 University of North Carolina at Chapel Hill, 107 W. Main Street, Apartment F, Carrboro, North Carolina 27510 USA; 4 Independent Leadership Consultant, Berlin, Germany; 5 Greater Accra Regional Hospital, Accra, Ghana; 6 Duke University School of Medicine, Durham, USA

**Keywords:** Maternal, Newborn and child health, LMIC, Leadership training, Leadership development, Coaching, Health workers

## Abstract

**Background:**

Frontline healthcare workers are critical to meeting the maternal, newborn and child health Sustainable Development Goals in low- and middle-income countries. The World Health Organization has identified leadership development as integral to achieving successful health outcomes, but few programs exist for frontline healthcare workers in low-resource settings.

**Methods:**

An 18-month pilot leadership development program was designed and implemented at Greater Accra Regional Hospital, a tertiary care facility in Ghana. A multi-modal training approach was utilized to include individual coaching, participatory discussions, role plays, and didactic sessions on leadership styles, emotional intelligence, communication, accountability and compassionate care.

**Results:**

A cross-section of 140 staff from 8 distinct hospital wards and 19 ranks were involved in various components of the leadership program from January 2014 to June 2015. At baseline, the primary leadership challenges and goals of the staff included: interpersonal communication, institutional logistics, compliance, efficiency and staff attitudes. Thirteen participants developed a total of 17 leadership projects to apply their training, many of which focused on improving challenges in organizational culture and systems through bettering leadership skills and interpersonal communication. The staff highly valued the program and found it beneficial to their work.

**Conclusions:**

Self-selected individual leadership projects mirrored areas of concern found in the needs assessment, indicating that the program was successful in achieving its goals. The on-site nature of the program was cost-effective and led to maximum staff participation despite clinical responsibilities. A longstanding relationship between the design team and the local hospital staff allowed for an exploration of approaches, many of which were new to the local context. Further research is needed on adapting the program to other settings in Ghana and integrating it into broader systems strengthening interventions. This pilot program was well received and warrants further adaptation and scale up.

## Background

Ending preventable maternal and neonatal death is at the core of the Sustainable Development Goals’ third objective of ensuring healthy lives and promoting well-being for all ages, where all children across the globe reach their fifth birthday and no woman dies as an outcome of pregnancy or childbirth. The vast majority of maternal and neonatal deaths occur in low-resource settings and many could be averted by strengthening health systems and processes through which care in pregnancy, during labor and delivery, and immediately postpartum are delivered [1, 2]. Leadership has been highlighted by the World Health Organization (WHO) as essential for strengthening health systems and improving health outcomes, and the WHO advocates for leadership development amongst health workers in low- and middle-income countries (LMIC) [3, 4]. Consensus is building that tackling current and emerging health problems must address the leadership capability of frontline healthcare workers in addition to strengthening clinical competency, organizational systems, monitoring and surveillance [5]. A growing body of evidence indicates a strong correlation between lack of leadership in clinical settings and poor patient outcome [6–8]. In maternal and newborn health, a recent Lancet review on health system bottlenecks lists the “absence of or weak supervisory, mentoring, and monitoring systems” as one of the critical gaps that contributes to neonatal death in LMIC [9]. Yet there are few examples of leadership development programs in low-resource environments that reach healthcare workers beyond upper-level managers.

Several leadership programs in low-income settings have been reported in peer-reviewed and other literature, such as the Senior Leadership Program at the National University of Rwanda School for Public Health for district hospital medical directors [10]; mentoring for academic leadership at Makerere University College of Health Sciences in Uganda [11]; and the Afya Bora Fellowship program in Botswana, Cameroon, Kenya, Tanzania, and Uganda for developing leadership skills of healthcare workers in training through online and in-person classes and mentoring [12]. Many of these programs focus on building capacity of those already in management positions in the health system, the Ministry of Health, or nongovernmental organizations (NGOs). Exceptions are the USAID-funded Leadership Development Programs (LDP and LDP+) for healthcare professionals in management and non-management roles, where evaluations from Brazil, Mozambique and Bangladesh report improvements in family planning and HIV testing outcomes [13]. In the peer-reviewed literature, a paper describing an LDP project that trained nurses in management, communication, and leadership skills successfully increased healthcare workers’ ability to identify and solve service delivery challenges, contributing to reduced maternal mortality rates in the target districts [14]. Overall, there is consensus on the need for leadership training of frontline staff in hospital settings and the promising evidence of its value.

This paper describes the design, implementation and evaluation of an eighteen month-long leadership training and coaching program for physicians, nurses, and midwives from January 2014 through June 2015 developed at a tertiary referral facility, Greater Accra Regional Hospital (GARH) in Accra, Ghana. The program was part of a multi-year partnership between GARH and Kybele, a non-profit humanitarian organization which promotes safe childbirth and compassionate care worldwide through collaboration with health professionals. GARH is one of the country’s largest obstetric referral centers within the Ghana Health Service, with a 90-bed maternity unit providing comprehensive care for more than 8000 annual births, the majority of which are transferred from peripheral facilities with complications. Ghanaian maternal mortality ratios (MMR) in regional and teaching hospitals such as GARH are reported as high as 957–1004/100,000 live births, though the national MMR is 319 [15–17]. The neonatal mortality rate in Ghana has declined from 39.5 to 28.1 per 1000 live births from 1990 to 2010, but further mortality reductions have stagnated [18]. The leadership program described in this paper is one component of an integrated approach developed by Kybele involving the simultaneous strengthening of clinical, operational and leadership capabilities for regional hospitals in Ghana. The elements of this overall approach, outcomes achieved, cost-effectiveness, and evaluation of clinical and operational strengthening activities are described elsewhere [19–22]. This paper focuses specifically on the leadership program.

## Methods

### Program concept

The program concept was developed based on discussions with the hospital management team responsible for maternal and neonatal health, observations of care by visiting clinical experts, and mortality audits. Observations revealed poor accountability regarding patient care, occasional verbal abuse, and poor communication with patients regarding labor progress, treatment plans, and newborn health status. The audit found avoidable leadership gaps associated with deaths at GARH, such as delays in calling a physician, inadequate patient surveillance and monitoring, and lack of consequences for poor performance leading to recurrent errors. The gaps identified were primarily in four areas that became the pillars on which the leadership program was based (Table 1): (a) adaptive decision making to solve problems; (b) personal accountability for the mother’s care and safety; (c) timely and appropriate communication with colleagues and patients and (d) compassionate care of the mother. At a high level, the leadership program was intended to bridge these gaps through targeted workshops and individual coaching, and an external leadership consultant from Europe trained in experiential coaching methods was engaged to deliver the program.

**Table 1:** Pillars of Leadership Development

Component	Definition in the GARH Context
Adaptive Decision Making	Doctors, midwives and nurses have the ability to adapt their decision making approach to the circumstances (e.g. deciding to break the established norm of waiting for a physician to visit the ward to examine a patient if the patent is in distress, or prioritizing a mother with an urgent need over someone who was ahead in the examination sequence).
Personal Accountability	Realization that every doctor, midwife and nurse has the obligation to be responsible for the patient’s safety and positive experience, and being ready to take the actions needed to exercise that responsibility.
Timely and Appropriate Communication	Comunication of appropriate information related to the care of the patient to each stakeholder (e.g. family member, colleague, or the patient herself) at the right time and in accordance with established protocols.
Compassionate Care	A cognitive, affective, and behavioral process consisting of: 1) Recognizing individual and universal suffering; 2) Feeling empathy for the person suffering and connecting with the distress (emotional resonance); 3) Tolerating uncomfortable feelings aroused in response to the suffering person (e.g. distress, anger, fear) so remaining open to and accepting of the person suffering; and 4) Motivation to act/acting to alleviate suffering.

### Needs assessment

Having established the major areas of concern through the audit, the program design was further customized through a comprehensive, participatory needs assessment to understand the leadership challenges that staff faced. Three leadership groups were established to provide input for the needs assessment, feedback on program design and ultimately participate in the majority of the program. The *Perinatal Leadership Forum* (PLF), consisting of physicians, nurses and midwives, was charged with championing leadership development across the organization. They met twice, forming a team charter to guide their work, and participated in the training sessions and individual coaching. Members of the PLF were interviewed to identify key leadership challenges and goals, and their responses were categorized into themes and later incorporated into training sessions. The other two groups were formed and designated as the *Leadership Ambassadors* and the *Clinical Champions*. Leadership Ambassadors were doctors and senior midwives responsible for bridging communication gaps between frontline staff and upper management. Clinical Champions were frontline midwives and nurses responsible for promoting leadership and quality within various clinical wards such as the labor and delivery unit, operating theatre, recovery room and newborn intensive care unit). Staff members in each group were carefully selected for their initiative and enthusiasm, and they represented all levels and ranks of the organization and all departments involved in maternal and neonatal care.

### Program design

**Fig. 1:**
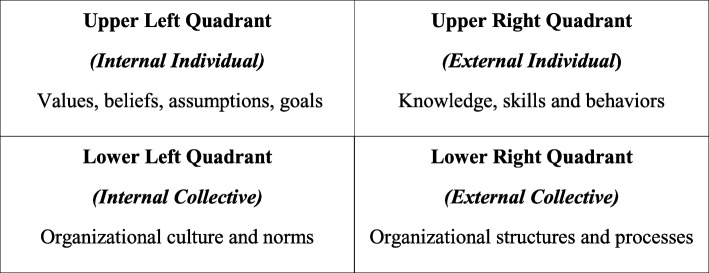
Four-quadrant exercise for individual coaching

**Fig. 2:**
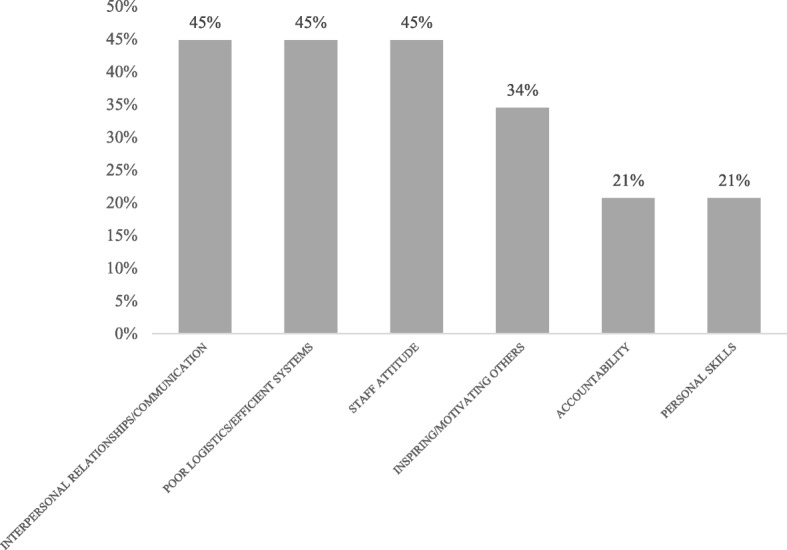
Leadership Challenges Reported During Needs Assessment (*n* = 27)

The needs assessment illustrated the leadership challenges most salient to the cross-section of healthcare providers at GARH and the goals they wanted to pursue during the leadership training. Some examples of the challenges reported are shown in Table 2. These were organized by Ken Wilber’s Four Quadrant Model of Integral Leadership [23] (Fig. 1) because it worked both to identify leadership challenges and then later to develop leadership projects. The four quadrants – Internal Individual, External Individual, Internal Collective, External Collective – have been adapted to analyze executive leadership by Volckmann [24], who interprets the Internal Individual quadrant as representing an individual leader’s values, beliefs, assumptions or goals that are often unstated or invisible; the External Individual quadrant reflecting leadership qualities that are visible, such as knowledge, words, actions or behavior; the Internal Collective quadrant taking into account the leader as part of an organization with a latent yet powerful culture and beliefs system; and the External Collective quadrant representing the organizational processes such as communication and reward systems that enable leadership to be influenced across the organization. Challenges were reported across all quadrants, indicating that the participants recognized the role of both individual and system issues affecting performance. Figure 2 shows the most frequently cited challenges, including interpersonal relationships and communication (fear of confrontation), poor logistics and inefficient systems, and staff attitude.

**Table 2:** Examples of Leadership Challenges by Quadrant of Wilber’s Model

Internal Individual	*Overcome fear of not being liked if exercising authority* *How to set boundaries* *How to inspire change* *Overcoming fear of conflict*
External Individual	*How to present ideas more convincingly* *How to run meetings more efficiently* *Improving ability to have “tough” conversations*
Internal Collective	*The lack of a culture of willingness to do things timely or appropriately* *Poor attitude of staff* *Team building and mobilizing staff about problem solving* *Establishing a strong system of accountability*
External Collective	*Improving emergency supply availability* *How to get better compliance with new practices and protocols* *Reduce separation time of mothers and babies after C-section*
Accountability	*Establish a strong system of accountability* *Ensure people feel accountable instead of blaming others* *Practice culture of accountability*
Personal Skills	*Time management* *Improve stress management skills*

**Fig. 3:**
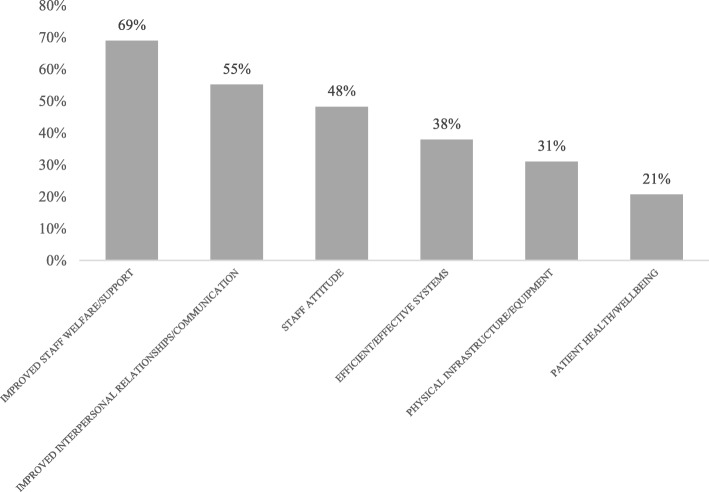
Leadership Goals Reported in Needs Assessment (*n* = 27)

The results from the needs assessment were used to refine and customize the program design to make it more relevant for the participants. The program still focused on the four key components of accountability, decision-making, communication and compassionate care. However, the scenarios developed for the workshops, and the examples used in the discussion and role-play sessions were based on the challenges and goals identified by the participants, and the coaching sessions were tailored to address the challenges and goals. Staff goals for the leadership program were ranked by frequency (Fig. 3) and organized by the Wilber model (Table 3) to determine the highest need. Overall, the priority for the leadership training was on improving staff communications and relationships, which was seen as a significant barrier to achieving good system performance and outcomes for patients. This goal mirrored the most commonly cited leadership challenges, which were related to individual and interpersonal communications.

**Table 3:** Examples of Leadership Goals by Quadrant of Wilber’s Model

Internal Individual	*Being more direct and “risk” not being liked* *Use different leadership styles for different situations* *Spend more time on wards building relationships*
External Individual	*Empower staff to be more accountable* *Mentoring and coaching staff to increase compliance and buy-in* *Show more appreciation* *Encouragement acknowledgement as a leverage for results*
Internal Collective	*Hold accountability meetings after negative outcome to coach responsible staff*
External Collective	*Map out flow of babies from wards to NICU* *Improve monitoring and documentation* *Redesign mortality audit form to include leadership gaps*

### Workshop design

During the needs assessment, members of the PLF were interviewed to identify key leadership challenges and goals, and their responses were then categorized into themes to be later incorporated into training sessions. These sessions commenced in January 2014 with multiple two-day workshops on emotional intelligence and Goleman’s Leadership Styles [25] held for the PLF, Clinical Champions and Leadership Ambassadors. The workshop began with a didactic presentation by the leadership consultant followed by a group discussion on emotional intelligence, its relevance to leadership, and an exploration of the Four Core Competencies of Leadership: self-awareness, self-management, social awareness, and social skills. Then followed a presentation and facilitated dialogue about Goleman’s six Leadership Styles (Coercive, Authoritative, Affiliative, Democratic, Pacesetting, Coaching) and their appropriate use. Participants role-played each style using examples of situations commonly encountered in everyday patient care. In April 2014, three “Accountability Workshops” were held for a cross-section of selected obstetrics and gynecology staff, including physicians, nurses, and midwives, some of whom were in the PLF and Leadership Ambassador groups and some of whom were general staff. The daylong workshop explored what a “culture of accountability” could look like at GARH and the accompanying structures to enact it, such as the establishment of accountability meetings whenever a death occurs in the maternity ward, moving from accepting system failures to active problem-solving to prevent them, and building practical skills for facilitating accountability dialogue between staff of different ranks to overcome hierarchical barriers to open conversations surrounding errors. Additionally, a series of six one-day workshops entitled “Compassionate Care” were offered in January and April 2015. These were intended for a broad range of staff to encourage an organization-wide shift in the norms towards compassionate care. The sessions focused on defining compassion and its importance to patient care to inspire more compassion towards one’s self, colleagues, and patients. Participants were taught how to skillfully and compassionately intervene when witnessing a colleague being disrespectful to another staff or patient. Participants were also given “Compassionate Care Champions” buttons and encouraged to wear them as a visible signal to patients and colleagues of their commitment to compassionate care. Finally, in September 2014 the Clinical Champions received a custom training on practical skills such as self-presentation, self-empowerment, listening, coaching and problem solving.

The teaching methods in the workshops were intentionally diverse and multi-faceted and included short didactic lectures, participatory role-playing, live coaching demonstrations, interactive group discussion, “appreciation circles” to build trust and community through shared gratitude and esteem for one another, personal reflection, small group feedback, and experiential storytelling exercises. Workshop participation was capped at 20 individuals to encourage interaction in small groups.

### Individual coaching

Workshops were coupled with individualized coaching sessions by the leadership consultant throughout the program. Staff involved in coaching volunteered themselves for coaching or were encouraged to participate by the leadership consultant based on observed responsibilities or responses during leadership training sessions. Coaching focused on identifying individual leadership challenges and developing leadership projects that were targeted to overcome these challenges. The Wilber Four Quadrant Model was again used because it allows for the opportunity to work on both individual and organizational leadership projects, thus allowing participants to select projects which were most relevant to them. With support through the coaching sessions, participants were encouraged to consider their priorities as leaders in their organizations, select a project that was meaningful and motivating to them, and apply the leadership skills they had learned in the workshops. The Wilbur model facilitated this well.

All leadership program activities were held on-site at GARH to facilitate wide participation and to lower program cost. No remuneration or per diem stipends were provided to staff for their participation.

### Program evaluation methods

A five-point questionnaire was administered at the conclusion of the program asking participants to assess the most personally valuable components of the program, the most meaningful teaching methods for learning and leadership development, the leadership challenges and priorities that were well addressed or not fully addressed, and their overall perceptions and feedback on the leadership program. Additional evaluation was conducted during the individual coaching sessions, asking participants in a one-on-one setting to report on their major takeaways from the program, specific challenges they aimed to address using new leadership skills, and progress towards achieving their leadership goals and objectives.

## Results

### Individual leadership project results

During the individual coaching sessions, participants were encouraged to take part in developing leadership projects that allowed them to apply the skills they learned in concrete ways. Thirteen participants developed projects primarily reflecting challenges in organizational culture and systems that they attempted to address through developing better individual and group leadership skills. The leadership consultant guided participants to develop specific action plans and goals for accomplishing their projects and followed-up in subsequent sessions to assess progress toward project achievement.

As indicated by the emerging fields of improvement and implementation science [26], leadership is necessary but not the only component for bringing about system change. However, selecting projects focused on organizational change as part of a leadership development program allowed the participants to test their leadership skills and to bring attention to system problems that had been previously ignored. Where challenges could be addressed by the direct application of skills learned, the participants put solutions in place. Where the challenges required system-level changes, the participants used their leadership skills to convene stakeholders and hold initial conversations about possible solutions, though the actual solution required combining leadership skills with the use of improvement and implementation science methods. A description of a sample of the projects, the leadership capability demonstrated, and the activities undertaken are shown in Table 4.

**Table 4:** Sample of Leadership Projects Developed during Individual Coaching Sessions

Project	Leadership Skill Practiced	Activities Undertaken
Start a Pregnancy School according to the requirements of the Ghana Health Service	Used the visionary and democratic leadership styles to present a vision and to involve others in its design	Conducted a stakeholder analysis and liaised with other hospitals who have developed a pregnancy school; built a leadership team for the school; presented a plan and timeline; designed a curriculum; collected or created teaching material.
Reduce the inflow of babies into NICU in order to decrease overcrowding and infection	Used the visionary, democratic, and facilitative leadership styles to engage staff in problem solving	Performed a stakeholder analysis and a value stream map to identify unnecessary admissions to the NICU
Ensure that mothers and babies are not separated after Cesarean delivery	Communication and accountability	Brought together three labor and delivery lead staff and the hospital director to coordinate a Babies-with-Mothers initiative for bringing babies to their mothers after a Cesarean delivery
Ensure the availability of hospital gowns such that all staff in the operating theatre wear proper hygienic attire and adhere to established protocols	Role played polite confrontation and exercised the behavior needed to gain compliance with protocol	Mobilized the GARH director and the Deputy Director of Nursing to support the plan, and successfully ensured gowns are consistently available. A meeting was also held with relevant staff to discuss hygienic attire protocols.
Improving availability of emergency supplies	Used the democratic leadership style to improve accountability and communication	Connected the procurement officer with head of finance to develop a system to reduce drug unavailability
Use motivation to manage increased workload and decreased staff numbers to improve efficiency	Used the visionary and democratic leadership style and role playing for decision making, accountability and communication	Made the morning meeting more concise and productive; called a meeting with surgeons towards improving better distribution of cases on morning, night and afternoon shifts; delegated tasks by sharing monthly night duty replacements among colleagues and handed over leadership of morning meetings by coaching others.
Ensure staff compliance with standard operating procedures (SOPs) in the operating theatre	Used the visionary and democratic leadership styles and one-on-one coaching for improved accountability and communication	Organized a meeting of staff and managers, mobilized the hospital director to convey the urgency of the matter and motivated staff to take ownership of the standard operating procedures.
Overcome challenges with noncompliant staff	Used appropriate authority with staff and more empowering leadership styles to inspire staff as warranted by the situation	Invested in a relationship with a midwife who was not complying with protocol, provided compassionate care, and coached her successfully on her attitude towards patients.

### Evaluation results

**Fig. 4:**
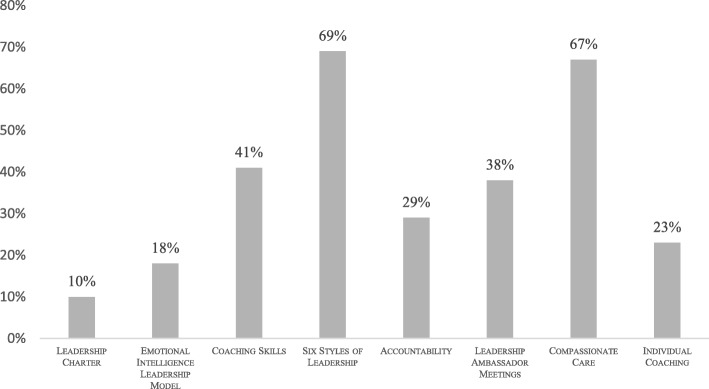
Program Components Ranked as Most Valuable by Participants (*n* = 20)

**Fig. 5:**
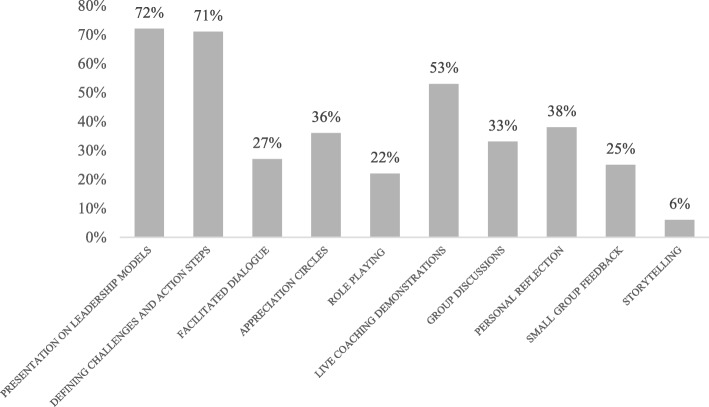
Leadership Teaching Methods Ranked as Most Valuable by Participants (*n* = 20)

A breakdown of the leadership program components and staff attendance can be found in Table 5. Of the 140 total participants, 27 were most heavily involved, having participated in the majority of the program. The five-question exit evaluation was intended for these 27 individuals and 20 were able to complete the questionnaire, with the remainder unable to do so due to scheduling conflicts. Respondents were asked to rank the program components and the teaching methods that they found to be most useful (Figs. 4 and 5). Figure 4 shows that the session focusing on Goleman’s Leadership Styles and the workshop on Compassionate Care were ranked as the top leadership program components by the largest number of participants. Sessions focusing on individual behaviors (accountability, emotional intelligence) or individual sessions (e.g. coaching) were not as highly ranked. The same pattern is apparent in Fig. 5. The most valuable teaching methods were presentations on leadership (highly ranked by 72% of participants), defining challenges and developing action steps (71%), and live coaching demonstrations (53%). Didactic training methods focused on concrete concepts, skills and tools were considered more useful than experiential ones that emphasized individual growth and change.

**Table 5:** Leadership Sessions by the Numbers

Leadership Session	Intended Group	Number of Attendees	Number of Total Sessions	Date of Sessions
Leadership Charter, Emotional Intelligence, Leadership Styles	Perinatal Leadership Forum	22	2	January 2014
Individual Coaching Sessions	Perinatal Leadership Forum	30	9	January, April, May, September 2014
Accountability	Perinatal Leadership Forum	22	3	May 2014
Leadership Ambassador Training	Leadership Ambassadors	6	1	September 2014
Clinical Champion Training	Clinical Champions	9	1	September 2014
Compassionate Care	All Staff	140	6	January and April 2014

Overall feedback on the participants’ experience with the leadership program was overwhelmingly positive. There was a consensus among staff that the program was effective in improving skills and that it should continue. Some sample comments, all of which were anonymous, included: “The program has built excellent leaders and managers and should be extended nationally for all health workers,” “The leadership program has given me confidence and skills to give effective care,” and “The program will go a long way to impact the lives of the staff.”

## Discussion

Overall, the leadership initiative at GARH proved to be a novel program that was well received. The topics of leadership style, emotional intelligence, compassionate care, and respectful confrontation were entirely new concepts for nearly all staff, and the participants engaged with the content and assignments with enthusiasm. The high level of participation and time commitment by so many staff, without remuneration, is noteworthy and indicative of the value they place on improving leadership, management, and accountability in their workplace. The needs assessment expressed a consistent message from across wards and ranks of the need for improvement in key areas—namely, holding people accountable while improving interpersonal communication, making improvements to logistics and processes, increasing staff compliance to protocols, and increasing positivity of staff attitudes.

The issues identified by staff as their most salient challenges closely correlated with the most highly ranked leadership goals, particularly improving interpersonal relationships and communication, improving staff welfare and support, and improving staff attitude. The leadership program aimed to address these key areas by using a mix of didactic and experiential learning, focused on personal reflection to develop individual leadership skills that could then be used to bring about improvements to the system.

This emphasis on building personal leadership skills meant that the program was designed to break down traditional hierarchical barriers between senior management and frontline workers in Ghana by involving both ranks in the same program. Management and staff were brought together in the same space to discuss individual behaviors and communication patterns and to learn styles of respectful confrontation to deal with situations affecting effective patient care. Participants eagerly engaged in these activities during the training.

The fact that a variety of program components and leadership teaching methods were highly ranked by participants indicates a need for further analysis in two areas. The first is a deeper assessment of the best contextual approach to build a sustainable leadership program to develop decision-making, accountability, compassionate care, and communication skills to improve maternal and neonatal mortality in tertiary facilities. This program reflected the approach of the leadership consultant who believed that changes to the system could come about when carefully selected staff worked diligently on their own thoughts, feelings and attitudes, and applied these new leadership behaviors to their everyday work. This is a time-consuming and resource-intensive process, which alone is not enough to tackle the types of projects that surfaced as priorities in Table 4. For example, it is not possible to reduce patient wait time or to ensure a consistent supply of drugs solely through personal leadership. The leadership training was therefore instituted as part of a broader system strengthening intervention that included clinical coaching and quality improvement training. The details of this program are described elsewhere [20] but integration of leadership and improvement and implementation science training in future designs is warranted.

Second, the approaches for “leveling the playing field” between higher and lower ranking positions in the organizational hierarchy to break down barriers in communication and interpersonal tension between staff is a countercultural practice in Ghana. Kybele had worked with GARH for several years prior to the introduction of the leadership program, and therefore had personal relationships with leadership and staff, which may have facilitated staff engagement in a program designed and led by a leadership consultant from a Western country. Further research is needed before transferring this approach to other settings. There are major differences in the way that these cultures view individuals in their workplace, with Ghanaians scoring high on *power distance* using the Hofstede model, meaning that “people accept a hierarchical order in which everybody has a place and which needs no further justification” [27] while Western Europeans and Americans score low on this leadership dimension, viewing hierarchy as inefficient and inconvenient. Similarly, Western Europeans and Americans score high on *individualism*, which indicates a high value placed on the individual and high expectation that one will look out for one’s self-interest. Ghanaians, however, score low on this dimension, suggesting higher value is placed on belonging to the collective and “taking responsibility for fellow members” [27] of one’s group – which may be an extensive network of immediate family, relatives and others. Before the program in the current form is expanded it is necessary to explore how these differences should be incorporated into a program that is adapted for the Ghanaian context. In addition, it is important to engage local leadership experts who are more likely to be attuned to cultural nuances related to the placement and exercise of power in Ghanaian organizational contexts.

Some additional limitations should be noted. The relatively short time period of the leadership program (18 months) resulted in limited analysis of the long-term impact of the program. Follow-up research is needed to illuminate the sustainability of skills learned by staff through the program and the achievement of leadership projects initiated. The focus of the program was on changing attitudes and behaviors; therefore, further time and efforts would be needed to show the impact of the program on specific maternal and child health outcomes. While participation in the program was impressively high, the time-constraints of staff members, who were juggling their clinical responsibilities with the added time commitment of the leadership sessions, may be another limitation in more extensive programming.

## Conclusion

Globally, designing and delivering leadership programs aimed at frontline staff remains a significant gap in LMIC. The pilot leadership program at GARH developed by Kybele showed that participants were highly interested in the topics of leadership development, which appeared relevant to key challenges in the workplace—both interpersonal and operational. Staff involvement in designing the interactive delivery model of the program resulted in a positive experience of the training and useful skills learned. The leadership projects provide further evidence of the staff's motivation in using the skills they have learned to address challenges in their work. There is a significant opportunity to build on the learning from this effort to develop a leadership curriculum. It was felt that reducing mortality and making more efficient use of resources required systematic improvement of service delivery processes in addition to strengthening leadership and therefore should not be undertaken as independent leadership projects isolated from other improvement activities.

Leadership development is clearly an essential component of quality healthcare and additional emphasis is warranted in LMIC to develop a new generation of leaders for the health system. This pilot research adds to the small but growing body of literature examining leadership programs that specifically target frontline healthcare workers in low-resource settings. The distinct aspects of this program included the multi-modal teaching methods (e.g. didactic, participatory, group workshops, appreciation circles, individual coaching) and the cross-sectional involvement of participants from the organizational spectrum of ranks and specialties. While the program described here has demonstrated the feasibility of developing a successful cost-effective leadership program for frontline health workers, future research in this very important area must be encouraged and undertaken.

## Abbreviations

DDNS: Deputy Director of Nursing Service
GARH: Greater Accra Regional Hospital
LDP+: Leadership Development Program Plus
LMIC: Low- and middle-income countries
MMR: Maternal mortality ratio
NGO: Nongovernmental organization
OB/GYN: Obstetrics and Gynecology
PLF: Perinatal Leadership Forum
QI: Quality Improvement
USAID: United States Agency for International Development
WHO: World Health Organization
